# The role of the macrophage-to-myofibroblast transition in renal fibrosis

**DOI:** 10.3389/fimmu.2022.934377

**Published:** 2022-08-05

**Authors:** Jia Wei, Zihao Xu, Xiang Yan

**Affiliations:** Department of Urology, Children’s Hospital, Zhejiang University School of Medicine, Hangzhou, China

**Keywords:** macrophage, macrophage-to-myofibroblast transition (MMT), renal fibrosis, myofibroblast, inflammation

## Abstract

Renal fibrosis causes structural and functional impairment of the kidney, which is a dominant component of chronic kidney disease. Recently, a novel mechanism, macrophage-to-myofibroblast transition (MMT), has been identified as a crucial component in renal fibrosis as a response to chronic inflammation. It is a process by which bone marrow-derived macrophages differentiate into myofibroblasts during renal injury and promote renal fibrosis. Here, we summarized recent evidence and mechanisms of MMT in renal fibrosis. Understanding this phenomenon and its underlying signal pathway would be beneficial to find therapeutic targets for renal fibrosis in chronic kidney disease.

## Introduction

Chronic kidney disease (CKD) affects around 11.7%–18% of the global population (1). While therapies can significantly delay the progression of CKD, the long-term prognosis remains worrisome (1, 2). Renal fibrosis is a dominant component of CKD, which is also the typical final stage of majority of the chronic and progressive nephropathies. It will eventually result in kidney failure (3). The renal fibrosis process consists of five steps, as follows (4): firstly, activation of renal tubular epithelia due to inflammation and infiltration of plentiful monocytes/macrophages into the kidney; secondly, excessive production of fibrogenic associated cytokines, growth factors, and other profibrogenic factors; thirdly, imbalance in the synthesis and degradation of the extracellular matrix (ECM) and the overdeposition and accumulation of ECM in the renal interstitium, which is a major stage of renal injury in structure and function; fourthly, mesenchymal changes in renal intrinsic cells and the decrease in the number of renal intrinsic cells; and finally, renal microangiopathy leading to ischemia and anoxia of the renal interstitium.

In renal fibrosis, activated myofibroblasts are a critical matrix-secreting cell type that plays a key role in ECM accumulation (5–7). Myofibroblasts are a heterogeneous population that may be derived from a variety of origins, including epithelia through epithelial-to-mesenchymal transition (EMT) (8, 9), endothelia through endothelial-to-mesenchymal transition (EndoMT) (10), and local fibroblast or pericyte proliferation (11). So far, literature indicates that monocytes/macrophages play an important role in the process of renal fibrosis, and the macrophage-to-myofibroblast transition (MMT) is recently discovered as another extrarenal genesis for myofibroblasts (12).

MMT is the transformation of macrophages into myofibroblasts in response to an inflammatory stimulation. The MMT cells are capable of producing collagen and are distinguished by the co-expression of a macrophage marker (CD68) and a myofibroblast marker [alpha smooth muscle actin (α-SMA)] (12). A study found that approximately 35% of the myofibroblasts in the unilateral ureteral obstruction (UUO) model of renal fibrosis originated from bone marrow cells (13). Another research discovered that 37% of myofibroblasts in the renal allograft were derived from the bone marrow (14). Thus, MMT acts a crucial role in the development of renal fibrosis. This review provides an update on current advancements in macrophages, with a particular emphasis on MMT-related mechanisms in renal fibrosis.

## Macrophages and macrophage-to-myofibroblast transition inrenal fibrosis

Previously, it is believed that macrophages contribute to renal fibrosis indirectly by secreting cytokines and chemokines that attract and proliferate fibroblasts. It has been well-documented that macrophages are phenotypic heterogeneous. According to various microenvironments, macrophages can polarize into a pro-inflammatory M1 phenotype or anti-inflammatory M2 phenotype. M2 macrophages could secrete interleukin (IL)-10 and transforming growth factor-β (TGF-β) to stimulate myofibroblast proliferation. Furthermore, M2 macrophages could also express procollagen I that contributes to fibrosis (15). Infiltrating macrophages are the major source of TGF-β in glomerulonephritis, and TGF-β plays a crucial role in the development of renal fibrosis (16). Increased *Ccl2* mRNA expression in infiltrating macrophages is associated with progressive renal fibrosis. The *Ccl2* gene encodes monocyte chemoattractant protein 1 (MCP-1), which is an emerging biomarker in acute kidney injury and a crucial chemoattractant regulator of macrophages in the renal inflammatory response. A prospective multicenter cohort clinical study reported that a higher level of MCP-1 was associated with significant estimated glomerular filtration rate decline and an increased incidence of CKD and CKD progression (17).

So far, literature has revealed that bone marrow-derived macrophages (BMDMs) can also directly contribute to renal fibrosis through MMT. Both *in vivo* and *in vitro*, infiltrating macrophages at the renal glomerular level could transition into myofibroblasts through the MMT process in diabetic nephropathy, which finally leads to renal fibrosis (18). Biopsy tissues from renal fibrosis patients showed that only in acute and active renal fibrosis does the number of CD68^+^α-SMA^+^ MMT cells correlate with the total α-SMA^+^ myofibroblast population. In acute inflammatory lesions, CD68^+^ macrophages were infiltrated, but few CD68^+^α-SMA^+^ MMT cells were found. In kidneys with advanced sclerosis, the number of α-SMA^+^ myofibroblasts was increased, but both CD68^+^ macrophages and CD68^+^α-SMA^+^ MMT cells were reduced. However, MMT cells were not found in normal human kidneys or those affected by minimal change disease (12). Depletion of myeloid lineage cells prevented the appearance of MMT cells and substantially reduced myofibroblast accumulation and collagen deposition (12).

## The origins of macrophage-to-myofibroblast transition in renal fibrosis

Urinary obstruction is a common cause of kidney fibrosis. Based on cell tracing in a murine UUO model, MMT cells were derived from bone marrow and were located in fibrosing mouse kidneys (19). The results found that more than 90% of monocytes/macrophages in the injured kidney came from bone marrow, while α-SMA was expressed in 22% of BMDMs, accounting for almost 80% of all α-SMA^+^ myofibroblasts in the injured kidney. Collagen I can be produced by active myofibroblasts, and the UUO mouse model revealed that about 70% of collagen I^+^ cells were transitioned from bone marrow monocytes/macrophages. Consequently, the infiltration of monocytes/macrophages into the injured kidney and the formation of a myofibroblastic phenotype are the principal contributions of cells to the development of renal fibrosis. In contrast, MMT cells were not detected in the kidneys of the sham-operated group. A study reported that BMDMs can develop into collagen-producing myofibroblasts during renal fibrosis in chimeric mice, supporting the idea that the myofibroblasts may originate from sources other than the renal system (13).

Renal chronic allograft rejection is also one of the major causes of renal fibrosis. BMDMs are a source of myofibroblasts in chronic allograft rejection and the development of renal interstitial fibrosis through the MMT process. Approximately half of the total myofibroblast population originated from BMDMs in human active chronic renal allograft injury. In patients with chronic renal allograft rejection, the number of CD68^+^ α-SMA^+^ MMT cells, not all myofibroblasts, was related to allograft functions and interstitial fibrosis severity (14). Lineage tracing in mice revealed that approximately 90% of the MMT cells originated in recipient bone marrow and contributed 37% of total myofibroblasts in the renal allograft. The results indicated that collagen-producing MMT cells accounted for an important component of the myofibroblast population in renal allograft injury and contribute to the development of fibrosis in renal allografts. It was established that the myeloid lineage of MMT cells comprised a significant proportion of the myofibroblast population and that the MMT process is a prevalent mechanism of renal interstitial fibrosis.

## Regulation of macrophage-to-myofibroblast transition and inflammatory signal pathways

**Figure 1:**
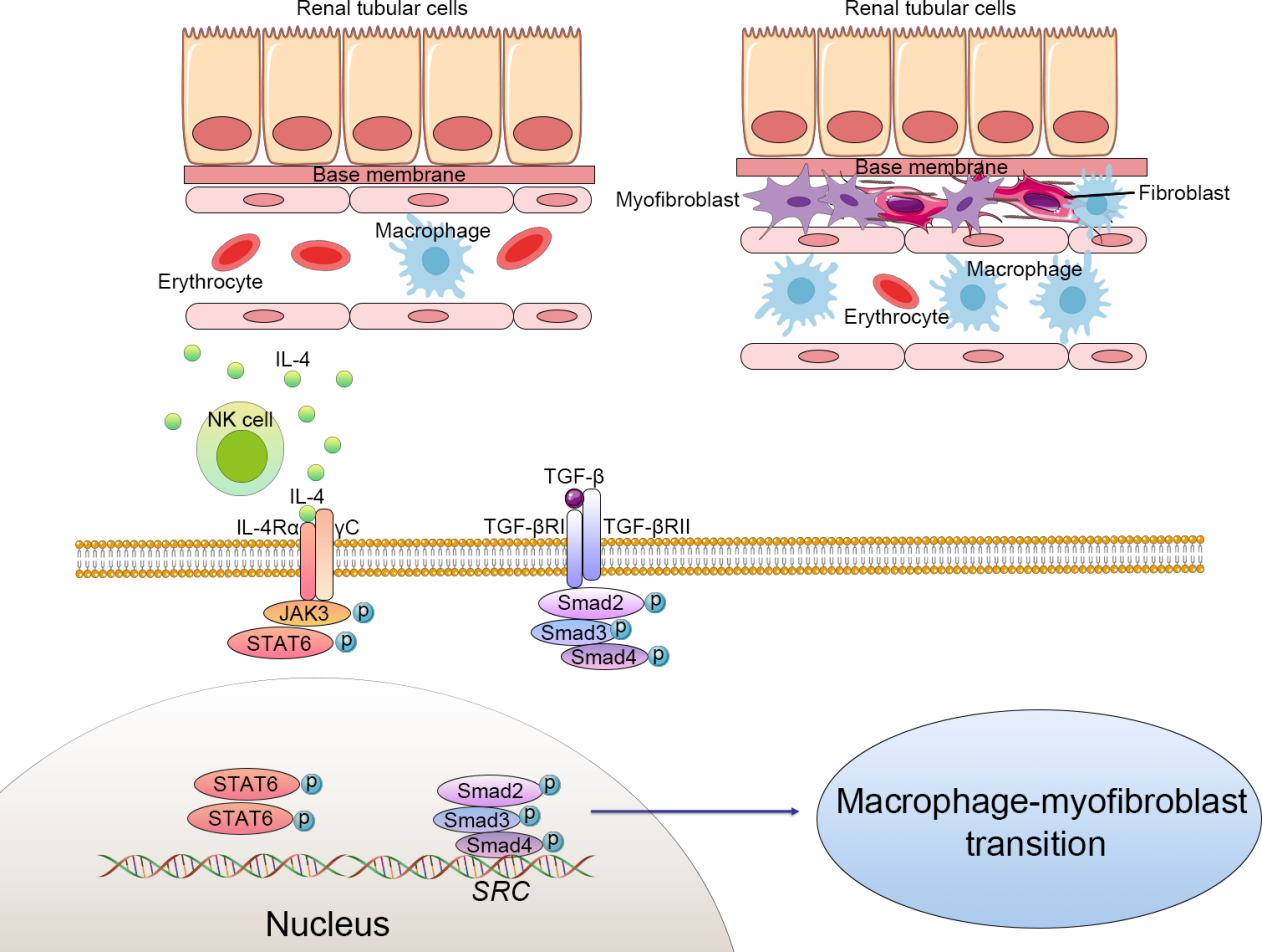
Cellular alteration and signaling pathway of macrophage-to-myofibroblast transition (MMT) in renal fibrosis. The macrophages derived from bone marrow differentiate to myofibroblasts, which produce excessive extracellular matrix and progressively cause structural and functional impairment of the kidney. Macrophage-to-myofibroblast transition plays a crucial role in renal fibrosis through multiple mechanisms, including triggering the transforming growth factor-β1 (TGF-β1)/Smad3 signaling pathways and natural killer T cells (NKT)/IL-4 signaling pathways. Activated NKT cells produce excessive IL-4, which subsequently combines with IL-4 receptor α and triggers the JAK3/STAT6 signaling to enhance the transformation of myofibroblasts. TGF-β binds to the TGF-β receptor complex and then phosphorylates the Smad family complex and finally activates the Src-centric gene network in bone marrow-derived macrophages to promote the MMT process. .

The classical signaling pathways in renal fibrosis include TGF-β1/Smad, nuclear factor-κB (NF-κB), Notch, Wnt, Hedgehog, phosphatidylinositol-3 kinase (PI3K/AKT), Janus kinase/signal transducers and activators of transcription (JAK-STAT), RHO/Rho coil kinase (ROCK), and tumor necrosis factor α (TNF-α) (20, 21). Some of these signal pathways are involved in renal fibrosis development by regulating the MMT process (**Figure 1**).

The TGF-β1-Smad3 signal pathway has been confirmed as the main mediator of the MMT process in BMDMs (14) (22). In brief, TGFR1 was activated by the interaction of TGF-β1 to its receptor TGF-β receptor 2 (TGFR2) (23). Activated TGFR1 induced the phosphorylation of Smad3 and Smad2, which then form complexes with Smad4. These complexes then enter the nucleus and activate the Src-centric gene network in BMDMs *via* transcriptional regulation, thereby promoting the MMT process in the fibrosing kidney. In this signaling pathway, Smad7 functions as a negative inhibitor, combining with activated TGFR1 and reducing the phosphorylation of Smad3 and Smad2. Smad7-deficient mice are more susceptible to renal fibrosis (24). The rerouting of TGF-β signaling from β-catenin/T-cell factor (TCF) to β-catenin/Forkhead Box O1(Foxo1) may affect the destiny of BMDMs. TGF-β signaling can be profibrotic or antifibrotic and anti-inflammatory, depending on which transfection factors are bound to β-catenin, TCF, or Foxo1 (25). An inhibitor of TCF (ICG-001) effectively suppressed the MMT process and reduced renal fibrosis. *In vitro* studies also showed that macrophage colony-stimulating factor (M-CSF) did not increase α-SMA and collagen I levels in Smad3^+/+^F4/80^+^ BMDMs. However, the expression of α-SMA and collagen Iα 1 could be detected in Smad3^+/+^ bone marrow macrophages incubated with TGF-β (26).

Smad3 is capable of directly binding specific Smad DNA-binding domains and promoting transcription, whereas Smad2 and Smad4 are transcriptional regulators but do not bind genetic elements. In the fibrotic kidney, the recruited Smad3^−/−^ macrophages failed to differentiate into myofibroblasts. This had a functional effect, as mice transplanted with Smad3^−/−^ bone marrow showed a remarkable reduction of collagen I deposition and α-SMA, indicating reduced renal fibrosis in the UUO kidney (26). Smad3 was required for the effective transition of recruited macrophages into collagen I^+^ α-SMA^+^ myofibroblasts in the injured kidney. Furthermore, the antifibrotic effect shown in Smad3^−/−^ chimeric mice suggests that BMDMs contribute to the development of renal fibrosis *via* MMT, while the process is mediated by TGF-β/Smad3 signaling. Using Smad3 inhibitors, such as a combination of asiatic acid and naringenin, could reduce the activation of TGF-β/Smad3 signaling and inhibit renal fibrosis (27).

The TGF-β1-Smad3 signal pathway plays an important role in regulating the MMT process in renal fibrosis. Researchers determined that POU Class 4 Homeobox 1 (Pou4f1), a direct Smad3 target gene in the TGF-β1-induced MMT process in BMDMs, was a potential therapeutic target to prevent renal fibrosis. It was found that Smad3 could bind to the promoter of Pou4f1 gene and enhance gene transcription. Then, Pou4f1 promotes MMT-mediated tissue fibrosis through a fibrogenic gene network. Pou4f1 silencing effectively inhibited MMT-induced tissue fibrosis *in vitro* and *in vivo* (28). Those processes in Smad3 mediating renal fibrosis were *via* miR-29 and miR-200 downregulation and miR-21, miR-129, and miR-210 upregulation (20).

Src is a tyrosine kinase that could be activated by numerous cytokines and growth factors [e.g., TGF-β1 and epidermal growth factor (EGF)], which has been identified to be associated with fibrogenesis (29). Inhibition of Src suppressed the activation TGF-β receptor and epidermal growth factor receptor (EGFR) and protected against renal fibrosis (30). Src is upstream of Smad3 signaling and acts as a direct Smad3 target gene, which is specifically upregulated in macrophages during MMT. Bioinformatic analysis indicated that Src is a key gene in the network of differentially expressed genes in TGF-β1-induced MMT (29). Smad3 can directly activate the tyrosine kinase Src and trigger collagen production, which inhibits the degradation of ECM, renal interstitial fibroblast activation, and renal fibrosis (30). Furthermore, activated Src has been reported to promote endocytosis and aggravate renal interstitial fibrosis (31). However, the Src inhibitor lacks specificity, which severely limits its application in the treatment of renal fibrosis.

JAK3/STAT6 signaling was involved in MMT. STAT6-deficient mice or mice treated with a JAK3 inhibitor (CP690,550) had fewer accumulations of bone marrow-derived fibroblasts in the experimental obstructed kidney model and developed less renal fibrosis. Treatment with a JAK3 inhibitor significantly affected the transformation of myofibroblasts, the production of matrix proteins, and the development of fibrosis in the obstructed kidneys (32). Recent studies have shown that STAT6 could suppress M2 macrophage polarization and be involved in the monocyte-to-fibroblast transition in folic acid nephropathy (33). Furthermore, STAT6-specific inhibitor (AS 1517499) has been proven to prevent M2 macrophage polarization and transformation of myofibroblasts, which lead to a reduction of collagen deposition and ECM production in the injured kidney (34). This evidence supports that JAK3/STAT6 signaling takes a crucial part in renal fibrosis *via* regulating the MMT.

In human kidney fibrosis, the majority of CD68^+^α-SMA^+^ MMT cells expressed CD206, indicating a predominant M2 phenotype (26). MMT and the bone marrow-derived fibroblasts are activated by natural killer T cells (NKT)/IL-4 signaling. As has been well-documented, IL-4 promotes macrophages toward M2 polarization, which plays a crucial role in bone marrow-derived fibroblast activation and renal fibrosis (35). According to research, IL-4Rα disruption decreased the number of CD45^+^α-SMA^+^ and CD206^+^PDGFRβ^+^ cells and renal fibrosis induced by renal obstructive injury. In addition, activation of NKT cells exacerbated the accumulation of MMT in the process of renal fibrosis. Furthermore, administration of IL-4 to CD1d-deficient mice increased bone marrow-derived myofibroblasts, promoted the MMT process, and developed a fibrotic process in the injured kidney (5).

Chemokines induced in cells of the injured kidney are responsible for the recruitment of circulating monocytes/macrophages. To suppress excessive BMDM-to-myofibroblast transition, therapy options could target chemokines and their receptors, such as chemokine ligand 2 (CCL2), CCL5, CCL21, C-X-C motif chemokine ligand 6 (CXCL6), CXCL16, and phosphatase and tensin homolog (PTEN) (36–39).

## Discussion and perspectives

In this review, we present the latest literature to identify that BMDMs promote renal fibrosis by both direct and indirect mechanisms. In a direct way, a part of BMDMs transition into myofibroblasts, which coexpress CD68 and α-SMA through MMT and produce collagen I to contribute to renal fibrosis. MMT plays a crucial part in the progression of renal fibrosis. The main signaling pathway of MMT is the TGF-β1-Smad3 signal pathway. Signaling-associated inhibitors had been proven to provide protection in animal models. The data suggest that MMT may be a therapeutic target for preventing renal fibrosis.

## Author contributions

JW and XY designed the article. JW and ZX drafted the article. All authors contributed to the article and approved the submitted version.

## Conflict of interest

The authors declare that the research was conducted in the absence of any commercial or financial relationships that could be construed as a potential conflict of interest.

## Publisher’s note

All claims expressed in this article are solely those of the authors and do not necessarily represent those of their affiliated organizations, or those of the publisher, the editors and the reviewers. Any product that may be evaluated in this article, or claim that may be made by its manufacturer, is not guaranteed or endorsed by the publisher.
